# “Management of andrological disorders from childhood and adolescence to transition age: guidelines from the Italian Society of Andrology and Sexual Medicine (SIAMS) in collaboration with the Italian Society for Pediatric Endocrinology and Diabetology (SIEDP)—Part-1”

**DOI:** 10.1007/s40618-024-02435-x

**Published:** 2024-08-10

**Authors:** M. Bonomi, B. Cangiano, S. Cianfarani, A. Garolla, D. Gianfrilli, F. Lanfranco, G. Rastrelli, E. Sbardella, G. Corona, A. M. Isidori, V. Rochira

**Affiliations:** 1 Department of Medical Biotechnology and Translational Medicine, University of Milan, Milan, Italy; 2 Department of Endocrine and Metabolic Diseases, IRCCS Istituto Auxologico Italiano, Piazzale Brescia 20, 20149 Milan, Italy; 3 Endocrinology and Diabetes Unit, Bambino Gesù Children’s Hospital, Rome, Italy; 4 Department of Systems Medicine, University of Rome “Tor Vergata”, Rome, Italy; 5 Department of Women’s and Children’s Health, Karolinska Institute, Stockholm, Sweden; 6 Unit of Andrology and Reproductive Medicine, Department of Medicine, University of Padova, Padua, Italy; 7 Section of Medical Pathophysiology and Endocrinology, Department of Experimental Medicine, “Sapienza” University of Rome, Rome, Italy; 8 Centre for Rare Diseases (Endo-ERN Accredited), Policlinico Umberto I, Rome, Italy; 9 Division of Endocrinology, Andrology and Metabolism, Department of Medical Sciences, Humanitas Gradenigo, University of Turin, Turin, Italy; 10 Department of Experimental and Clinical Biomedical Sciences “Mario Serio”, Careggi Hospital, University of Florence, Florence, Italy; 11 Endocrinology Unit, Medical Department, Maggiore-Bellaria Hospital, Azienda Usl, Bologna, Italy; 12 Endocrinology, Department of Biomedical, Metabolic and Neural Sciences, University of Modena and Reggio Emilia, Modena, Italy; 13 Unit of Endocrinology, Department of Medical Specialties, Azienda Ospedaliero-Universitaria Di Modena Policlinico Di Modena, Ospedale Civile Di Baggiovara, Via Giardini 1355, 41126 Modena, Italy

**Keywords:** Transitional age, Transition guidelines, Transition andrology, Andrological diseases, Adolescence andrology, Childhood andrology

## Abstract

**Purpose:**

Andrological pathologies in the adulthood are often the results of conditions that originate during childhood and adolescence and sometimes even during gestation and neonatal period. Unfortunately, the reports in the literature concerning pediatric andrological diseases are scares and mainly concerning single issues. Furthermore, no shared position statement are so far available.

**Methods:**

The Italian Society of Andrology and Sexual Medicine (SIAMS) commissioned an expert task force involving the Italian Society of Pediatric Endocrinology and Diabetology (SIEDP) to provide an updated guideline on the diagnosis and management of andrological disorders from childhood and adolescence to transition age. Derived recommendations were based on the grading of recommendations, assessment, development, and evaluation (GRADE) system.

**Results:**

A literature search of articles in English for the term “varicoceles”, “gynecomastia”, “fertility preservation”, “macroorchidism”, “precocious puberty” and “pubertal delay” has been performed. Three major aspects for each considered disorder were assessed including diagnosis, clinical management, and treatment. Recommendations and suggestions have been provided for each of the mentioned andrological disorders.

**Conclusions:**

These are the first guidelines based on a multidisciplinary approach that involves important societies related to the field of andrological medicine from pediatric to transition and adult ages. This fruitful discussion allowed for a general agreement on several recommendations and suggestions to be reached, which can support all stakeholders in improving andrological and general health of the transitional age.

**Supplementary Information:**

The online version contains supplementary material available at 10.1007/s40618-024-02435-x.

## Introduction

Many of the andrological pathologies that andrologists face in their adult patients, concerning genital, hormonal, reproductive, oncological, sexual, and psychological aspects are the results of conditions that originate during childhood and adolescence and sometimes even during gestation and neonatal period. For this reason, andrology is the medical specialty that must deal with men’s health and reproductive function not only during adulthood but more correctly from birth, or even gestation, to ageing of male subjects. Indeed, it is well known that male gonads, together with the hypothalamic GnRH-secreting neurons and the pituitary gonadotroph cells are extremely sensitive to many different factors (i.e. genetic, environmental, chemical, physical, etc.… events) that can influence their correct development and functionality during gestation, neonatal age up to puberty and transitional age [1–3]. Therefore, the role of the andrologist is extremely important not only for adult but also pediatric patients. Indeed, an andrological examination should be recommended routinely to all male subjects both in the prepubertal and post-pubertal period. The andrological evaluation of neonatal, pediatric, and adolescent patients is fundamental in acquiring an early diagnosis of many andrological disorders such as disorders of sexual development, structural disease of the genital organs, normal and abnormal puberty, undescended testes, genital tumors, gonadal function, and its relationship to growth, virilization, fertility and gender identity. These pathologies, if not diagnosed with routine screenings, could affect prepubertal, pubertal and post-pubertal subjects, and a different approach would be needed for each age range. Frequently observed disorders are cryptorchidism, varicocele, hydrocele and funicular cysts, inguinal hernia, phimosis, and hypospadias. Less frequently observed disorders are orchitis-epididymitis (i.e. mumps), testicular torsion, microlithiasis, Leydig cells hyperplasia and gynecomastia. Rare observed disorders are gonadal cancer, hypogonadism, and ambiguous genitalia.

Although in the last 5 years an increasing number of papers looking foetal programming and minipuberty have been published, the reports in the literature concerning other pediatric andrological diseases are not so copious and they are mainly concerning single issues [4–12].

Aim of the present paper was to analyze the state of the art regarding the clinical evidence on several different andrological disorders affecting pubertal, and transitional age and to provide a shared position statement of the Italian Societies for Andrology and Sexual Medicine (SIAMS) in collaboration with the Italian Society for Pediatric Endocrinology and Diabetology (SIEDP). Three major aspects for each considered disorder were assessed: diagnosis, clinical management, and treatment. A Part 2 of these guidelines will follow with coverage of other specific conditions such as DSD, micropenis, hypospadias, cryptorchidism, epidydimitis-orchitis, testicular torsion, testis cancer.

## Material and methods

A literature search of articles in English for the term “varicoceles”, “gynecomastia”, “fertility preservation”, “macroorchidism”, “precocious puberty” and “delayed puberty” has been performed. In this guideline, we will provide recommendations regarding the evaluation and management of different andrological disorders during childhood and transitional age based on the GRADE (grading of recommendations, assessment, development, and evaluation) system for grading both the quality of evidence and the strength of recommendations [13] According to this system, the strength of recommendation will be divided into ‘strong’, indicated by the number 1 and ‘we recommend’, and ‘weak’ indicated by the number 2 and ‘we suggest’. The grading of the quality of evidence is denoted as follows: ⊕ ○○○ for very low-quality evidence; ⊕  ⊕ ○○ for low quality; ⊕  ⊕  ⊕ ○ for moderate quality; and ⊕  ⊕  ⊕  ⊕ for high quality [13].

### Varicocele

#### Epidemiology

Varicocele prevalence in paediatric age widely ranges from 4.0% to 35% with an age-dependent increase. In boys younger than 10 years, prevalence is < 1% [14–16]. Prevalence starts rising at age 11–12 years [14, 17] and peaks in late adolescence varying between 14% [14] and 27% [17, 18]. In studies from the general population, most boys have a varicocele which is palpable with Valsalva maneuver (corresponding to Dubin’s grade 1 as described below) [17]; in urological studies, a varicocele which is already visible at the scrotum physical examination without Valsalva maneuver (corresponding to Dubin’s grade 3 below described) is much more frequent (up to 70%) [19].

#### Pathophysiology

On the left side, the spermatic vein drains into the renal vein at 90° angle, which is a predisposing factor for varicocele. Age is a predictor probably because of the rapid growth occurring during puberty [16, 20]. Accordingly, taller, and leaner boys are at higher risk [16, 20]. Lower testis volume, greater penis length and girth are further statistically significant predictors [16, 20]. However, a clear pathogenic mechanism was not identified for these. Inheritance has a role only for higher graded varicocele and no relationship was found for familiar history of varicose veins [21]. Longitudinal studies assessing the natural history of untreated varicocele in terms of fertility are limited, biased and conflicting [22–24].

#### Clinical picture

Varicocele can be subclinical (i.e., detected only by ultrasound) or clinically detectable, palpable as a “bag of worms”. Only rarely, it is painful (3%) [19]. Almost 90% of varicoceles are left-sided, around 10% are bilateral, and only a minority are right-sided [14]. Right-sided varicoceles in children and adolescents may be a rare sign of Wilms’ tumour [25].

#### Diagnostic evaluation

##### Suggestions and recommendations

**R1.1** We recommend assessing varicocele by physical examination in recumbent and standing position. (1, ⊕  ⊕  ⊕ ○).

**R1.2** We suggest using color Doppler ultrasound to further characterize and grade the varicocele. (2, ⊕  ⊕ ○○).

**R1.3** We recommend using ultrasound to accurately assess testicular hypotrophy or asymmetry. (1, ⊕  ⊕ ○○).

##### Evidence

According to Dubin’s classification, grade I varicocele is palpable with Valsalva maneuver, grade II is palpable without Valsalva, grade III is visible without Valsalva [26].

Testicular ultrasound might be helpful in confirming and better characterizing the varicocele [27]. It allows the exact evaluation of the varicose veins’ extension and dilation according to Sarteschi/Liguori scoring system [28, 29].

Testicular volume or semen analysis, as surrogate markers of future fertility, have been assessed by cross-sectional or short-term retrospective studies. Data on the effect of varicocele on testis volume are conflicting with studies reporting no [30] or small [31] effect and other reporting a progressive decrease for Dubin’s grade 2 and 3 varicoceles [32]. Testis volume asymmetry > 20% has been associated with significant impairment in semen parameters (66% with impaired sperm motility) [33].

##### Remarks

Varicocele assessed either by physical examination or color Doppler ultrasound must be performed in recumbent and standing position with Valsalva maneuver. Ultrasound may be used for confirmation, as only palpable varicoceles deserve treatment. The Sarteschi/Liguori classification divides varicoceles in five grades, depending on presence of varicosities. Grade IV and V of Sarteschi/Luguori classification correspond to Dubin’s grade III. Scrotal ultrasound may be useful for testis volume assessment—using Lambert’s formula (length*width*height*0.71)—since, in adolescents, clinical evaluation could be inaccurate in detecting hypotrophy [34–36]. Evidence on semen analysis in adolescents with varicocele is still limited.

#### Therapeutic management

#### Suggestions and recommendations

**R1.4** We suggest offering treatment in adolescents with testicular hypotrophy or asymmetry (difference > 20%). (2, ⊕  ⊕ ○○).

**R1.5** We suggest considering pain and discomfort for decision making about varicocele treatment, despite evidence is limited. (Expert opinion).

**R1.6** We suggest lymphatic sparing surgery or percutaneous embolization, although, at present, no technique has demonstrated to be better. (2, ⊕  ⊕  ⊕ ○).

**R1.7** We suggest only monitoring for patients not candidate for surgical treatment. (Expert opinion).

##### Evidence

A recent meta-analysis of the available randomized clinical trials (RTCs) [37] shows that surgery corrects varicocele in more than 85% of cases. As compared with clinical observation, intervention is associated with slightly increased testis volume and higher total sperm count [37]. However, only four and two RCTs were available for these outcomes, respectively [37]. There is not enough evidence for drawing conclusions on future paternity [37]. Pain was rarely assessed; however, treatment resolved it in > 90% of cases [37].

There is not enough evidence for indicating which is/are the best surgical technique/s to use in adolescents [37]. Since the most common adverse event is hydrocele and this is limited by lymphatic sparing surgery, this should be preferred [37]. Percutaneous embolization techniques may be an alternative to surgery and radiological risk should be considered especially for younger patients [38, 39].

##### Remarks

Although conflicting and limited, present evidence suggests that treatment of varicocele should be offered in adolescents with hypotrophy or asymmetry > 20% of testis volume [32, 33, 40–42]. However, this could lead to overtreatment since asymmetry is a frequent finding also in adolescents without any scrotal disease [43]. In case of conservative approach, the clinical follow up should include periodical physical examination, ultrasound, and semen analysis. The latter should be considered with caution and limited to patients with Tanner stage V. Asymmetry worsening over time may be more informative. Follow-up studies suggest that a catch-up grow in testicular volume of treated adolescents and young adults can occur [44]. However, the evidence on the natural history of untreated varicoceles in adolescence is lacking. Since regular monitoring of subclinical varicoceles is suggested in adulthood [45], a similar approach might be a good clinical practice also in adolescence [44].

### Gynecomastia

#### Epidemiology

Gynecomastia is the benign enlargement of the male breast caused by proliferation of glandular tissue [46]. It can be unilateral or bilateral, most commonly the latter [47, 48]. It is the most common breast alteration in males, being mostly physiologic during infancy, puberty, and old age [49, 50]. Prevalence rates are 65–90% in newborns, 22–69% in adolescents, and 24–65% in men between 50 and 69 years [51, 52].

#### Pathophysiology

The causes of gynecomastia are multifactorial, and 25% of all cases appear idiopathic. Among the underlying causes, three groups of triggers are distinguished: physiologic, pathologic and pharmacologic/toxic [50, 52].

The peak prevalence in adolescents is observed during mid-puberty, when the sex hormones surge [53]. Underlying endocrinopathy cannot be detected in most cases, and spontaneous regression can be expected within 6 months or less but may persist up to 1–2 years [54].

#### Clinical picture

Gynecomastia of infancy can persist for several weeks after birth and can cause mild breast discharge called “witch’s milk” [46]. Gynecomastia of infancy is not associated with any sequels or aberrations of development; typically, it does not persist after the first year of life. Detection of glandular breast tissue requires a careful physical examination feeling a firm, rubbery, finely lobular mobile disc of tissue that extends concentrically from under the nipple and areola. Although it is mostly bilateral, it is often asymmetrical and can occur unilaterally. Psychological or sexual issues at puberty are among the chief complaints, although pain may be frequent [51].

#### Diagnostic evaluation

##### Suggestions and recommendations

**R2.1** We suggest that the initial screening to rule out lipomastia, obvious breast cancer, or testicular cancer might be performed by a pediatrician, a general practitioner, or another non-specialist. (2, ⊕  ⊕ ○○).

**R2.2** We recommend that in those cases where a thorough diagnostic workup is warranted, it should be performed by a specialist in endocrinology/pediatric endocrinology/andrology. (1, ⊕  ⊕  ⊕ ○).

**R2.3** We recommend testis and breast US to rule out possible tumors. (1, ⊕  ⊕  ⊕ ○).

##### Evidence

The primary goal of the initial evaluation should be to confirm the presence of palpable glandular tissue and rule out the suspicion of malignant breast tumor or testicular tumor. In the setting of simple lipomastia, the condition can be diagnosed and followed up by palpation; the finding of palpable glandular tissue suggest the need for breast and testicular US [51]. Persistence during adolescence or a new and rapidly developing condition may warrant further workup [52]. To rule out malignant tumors, breast (ultrasound and mammography) [55] and testis (ultrasound) imaging is mandatory [56].

Andrological history should include information on cryptorchidism, the onset of puberty, and symptoms of testosterone deficiency. Information on general illness, use of medications, use of anabolic–androgenic steroids (AAS), alcohol, cannabis, and drug abuse should also be noted [57]. Thyroid disorders should also be ruled out.

If gynecomastia is mild and non-progressive or if adolescent gynecomastia, according to Tanner stage, is a very likely diagnosis, the initial laboratory evaluation comprises serum early-morning (i.e., before 10 AM) total testosterone, estradiol, LH, and FSH levels. In less straightforward cases, the basic laboratory workup should include also TSH, FT3, FT4, SHBG, prolactin, β-hCG, and liver and renal function tests [46, 49–51, 57].

##### Remarks

To which degree pubertal gynecomastia needs diagnostic workup is controversial; in the expert hands, it can be restricted to physical examination [47]. The initial screening of gynecomastia might be performed by a pediatrician or a general practitioner to rule out the presence of mammary or testicular cancer. The workup of gynecomastia should be carried out by a specialist in endocrinology/pediatric endocrinology/andrology.

#### Therapeutic management

##### Suggestions and recommendations

**R2.3** We do not recommend medical therapy for pubertal gynecomastia, which resolves spontaneously within 24 months in more than 90% of cases. (1, ⊕  ⊕ ○○).

**R2.4** We recommend withdrawal of interfering drugs, whenever feasible, treatment of concomitant disorders, and discontinuation of substances of abuse or inducing drugs, before considering a specific treatment for gynecomastia. (1, ⊕  ⊕  ⊕ ○).

**R2.5** We suggest surgical treatment only for patients with long-lasting gynecomastia, which does not regress spontaneously, or which causes considerable psychosocial and psychological distress, after puberty completion. (2, ⊕  ⊕ ○○).

##### Evidence

In cases of gynecomastia of puberty (or adulthood) with negative physical and hormonal investigations, there is a fair chance that the condition will disappear spontaneously, especially if it is of recent onset [47, 54].

If gynecomastia is severe, does not resolve, and does not have a treatable underlying cause, some medical therapies may be attempted. Data on pharmacologic therapy for pubertal gynecomastia is partial and scarce, with insufficient high-quality evidence-based original research on efficacy and safety in transitional age. Pharmacological treatment can have an advantage in relieving behavioral and psychological distress [58]. There are three classes of medical treatments: androgens, anti-estrogens and aromatase inhibitors. Tamoxifen has been used in gynecomastia of puberty with partial response in most cases (90%) but a complete response in < 10% [58, 59]. Gynecomastia in Klinefelter syndrome boys, which incidence (35.6%) is not increased compared with typically developing boys, can be reduced or resolved by testosterone supplementation, potentially preventing the need for surgery [52].

When medical therapy is ineffective, particularly in cases of longstanding gynecomastia, when the breast enlargement is severe, painful, socially embarrassing, or when there is suspicion of breast malignancy, then surgical therapy is appropriate [50, 60].

##### Remarks

Surgical treatment should not be offered until after an observation period has been allowed [47]. Surgical treatment should be postponed preferably until after completion of puberty to minimize the chance of recurrent gynecomastia after surgery. In cases of pubertal gynecomastia where the disease causes considerable psychosocial and psychological distress, surgical treatment is justified [60, 61].

Particular attention should be paid to gynecomastia in boys of prepubertal age, a rare finding, which is not anticipated by normal hormone fluctuations and warrants thorough evaluation to rule out an underlying pathology [46].

### Fertility preservation

From childhood and adolescence to transition age, some categories of male patients should receive counseling related to fertility preservation (FP). These categories are: (i) patients who are candidates for potentially sterilizing therapies; (ii) patients with Klinefelter syndrome (KS) and other aneuploidies; (iii) subjects with disorders of sex development (DSDs); (iv) transgender persons assigned male at birth (T-AMAB) undergoing gender affirming treatments.

#### Epidemiology

i) Many types of cancers, both solid and hematologic, and more rarely immunological and rheumatological conditions of adolescents, can affect males during the transitional age. The incidence of childhood cancer has steadily increased since the 1950s [62] and the incidence in children aged 0–14 is reported of 140.6 per million person/year [63].
ii) KS (47,XXY syndrome) and other chromosomal abnormalities leading to male infertility, such as the presence of more than two supra-numerous X chromosomes, or different types of mosaicisms, occurs in about 152–223 cases per 100,000 males and is the most frequent (numerical) chromosomal disorder in males and, although nowadays the greater use of prenatal diagnosis techniques is allowing ever-increasing percentage of early diagnoses, it is currently often diagnosed in adulthood [64–66]. KS is one of the most frequent causes of infertility, affecting 10–15% of azoospermic infertile men [67].
iii) DSD is a group of congenital conditions characterized by atypical development of chromosomal, gonadal or anatomic sex that leads to a discordance between the genital appearance and the chromosomal sex and infertility [68, 69]. DSD worldwide incidence is approximately 1:1000–4500 live births. Excluding simple cryptorchidism and hypospadias the incidence rate among subjects with a 46,XY karyotype to have a DSD has been estimated to be 1 in 20,000 births [70].
iv) The prevalence of referral gender incongruence is increasing worldwide [71]. A recent demographic report shows that over 1.6 million people identify themselves as transgender in the United States (0.6% of population), and they account for 1.4% of young people between 13 and 17 years old [72, 73]. Fertility preservation represents an increasingly crucial aspect of healthcare nowadays for T-AMAB individuals who want to become women and at the same time who wish to have their own biological offspring in the future.

#### Pathophysiology

i) Young males who undergo cancer treatments (chemo/radiotherapy or surgical treatments affecting the genital tract) are more frequently affected by infertility than females of the same age [74]. It has been estimated that one third of cancer survivors treated at prepubertal age will be azoospermic at adult age while one fifth will be oligozoospermic [75–77]. So far, there are no data available on the future fertility of subjects treated for immunological and rheumatological pathologies in adolescence.
ii) Boys with KS progressively lose their spermatogenic capacity. From early puberty to mid-puberty, there is a histological change starting with relatively normal seminiferous tubules, reduced germ cells, and normal Sertoli/Leydig cells to the adult condition showing extensive fibrosis and hyalinization of the seminiferous tubules [78]. Up to 8% of men with KS have spermatozoa in the ejaculate [79, 80] and in about 60% the sperm recovery can be obtained through testicular sperm extraction [81, 82]. US imaging can help in identifying progressive time and puberty-related deterioration of testicular structure [83].
iii) Infertility in DSD can be due to anatomical defects, abnormal gonad development, prophylactic gonadectomy and hormone replacement therapy to induce and sustain puberty [84].
iv) Gender affirming hormonal therapies (GAHT), which are an integral part of gender transition in T-AMAB, have a significant impact on fertility [85]. Moreover, gender affirming surgery (GAS) usually leads to permanent sterility in these patients [71].

#### Clinical picture

The clinical presentation can be different according to the patient’s age and issue. Unlike post-pubertal youth, childhood subjects have not yet reached spermarche and therefore have immature testicles presenting only spermatogonial stem cells. Cancer patients and T-AMAB usually have testicles that function normally while subjects affected by KS and DSD have always dysgenetic testicles with altered architecture of the seminiferous tubules.

#### Diagnostic evaluation

##### Suggestions and recommendations

**R3.1** We recommend testicular physical examination and pubertal stage evaluation in all pediatric patients to guide towards the appropriate FP option. (1, ⊕  ⊕  ⊕ ⊕).

**R3.2** We suggest providing adequate counseling on FP to these patients and their parents. (Expert Opinion).

##### Evidence

More studies suggest that in case of risk of gonadal damage and fertility loss, patients should be referred to the infertility specialist at diagnosis and before gonadotoxic treatments [86, 87]. The updated guidelines emphasize the importance of addressing gonadotoxicity and FP in all patients with reproductive potential, including the pediatric population [62]. Parents need to be made aware of and be receptive to fertility preservation options while young patients must also be receptive to discussions about fertility preservation, suitable to their age [88–90].

##### Remarks

It is essential that the clinical team has a detailed history and knowledge of the hormonal pattern, testicular physiology, and pubertal stage [87] to choose the appropriate protocol of FP. Early conversations should be initiated at the time of diagnosis to provide patients/parents with accurate information regarding FP options and to maximize the number of strategies available to the patient [62, 91].

#### Therapeutic management

##### Suggestions and recommendations

**R3.3** We recommend sperm cryopreservation in all young boys able to collect semen and in which it is possible to retrieve mature sperm. (1, ⊕  ⊕  ⊕ ⊕).

**R3.4** We suggest discussing testicular biopsy with tissue banking in case of prepubertal or azoospermic subjects. (2, ⊕  ⊕ ○○).

##### Evidence

Cryopreservation of semen is a well-established method for fertility preservation in post-pubertal males and in patients before gonadotoxic treatments [92, 93]. For boys who have not yet reached spermarche, investigational options provide significant hope for the future. These techniques rely upon the testicular biopsy and cryopreservation of either whole tissue or spermatogonial stem cells (SSCs). While testicular biopsy is considered safe and effective for adults, its use for prepubertal patients is currently considered experimental and its suitability should be discussed very carefully with the treatment team on a case-by-case basis [94–98].

##### Remarks

Sperm cryopreservation is a very valuable approach that should be performed in all boys who are able to collect sperm and who are at risk of infertility or undergoing potentially sterilizing therapies [94, 99].

For pre-pubertal subjects who cannot produce an ejaculate, or patients who do not have sperm in their semen, a piece of testicular tissue can be surgically removed to be frozen and stored [62, 87]. Although conflicting and limited, present evidence suggests that future advances in the use of cryopreserved testicular tissue will represent the only fertility chance for these males facing sterilizing conditions [96–98, 100, 101]. However, this option should be considered experimental and there is no guarantee that the stored sperm or testicular tissue will enable subject to father in the future [62].

### Macroorchidism

#### Epidemiology

Macroorchidism is a condition that describes an increase of testicular volume, extremely rare in children and adolescents and usually included in the clinical presentation of some genetic syndromes or endocrine disorders.

#### Pathophisiology

Several genetic syndromes have been associated with macroorchidism, but the most frequently described in literature are the X-fragile syndrome (caused by a CGG trinucleotide repeat expansion in the *FMR1* gene*)* [102] and the loss-of-function mutations in immunoglobulin superfamily member 1 (*IGSF1*) gene. This latter gene deficiency causes specific impairment of pituitary secretion which can be responsible of testicular enlargement, such as central hypothyroidism (with prolonged proliferation of Sertoli cells) and high FSH values [103, 104]. Other X-linked syndromes in which macroorchidism is described are the Clark-Baraitser syndrome [105] and the Atkin-Flaitz syndrome [106]. Among autosomic genetic disorders, McCune-Albright [107–109] and Beckwith-Wiedemann syndrome [110] have been associated with macroorchidism.

Apart from genetic conditions, also some rare endocrine disorders can present with macroorchidism such as FSH-secreting pituitary adenomas [111, 112], congenital adrenal hyperplasia (CAH) [113] and severe aromatase deficiency [114]. Finally, in this context, it is important to early identify testis enlargement due to testicular tumors or lymphomas with testicular localization [115].

#### Clinical picture

Macroorchidism can be unilateral or bilateral. Testicles are usually enlarged (above + 2SD curve for age) [116] with normal consistency (unless the cause is testicular cancer). Bilateral macroorchidism increase the suspicious of genetic and endocrine syndromes but also, in some cases, lymphomas [115]. Sometimes McCune-Albright syndrome can also present with unilateral enlargement [108]. In fragile X syndrome, patients have significantly larger testes throughout childhood, with true macroorchidism becoming manifested usually just prior to puberty [116–118] while it is always post-pubertal (or even with adult onset) in IGSF1 deficiency [119].

A sudden enlargement of the testicles may depend on testicular tumors, lymphomas or FSH-secreting pituitary adenomas [111, 112, 115, 120].

The association with other signs typical of genetic syndromes can help in differential diagnosis. Fragile X syndrome is usually characterized by intellectual disability and a characteristic phenotype [117, 121]. For this reason, the diagnosis is often made around 2–3 years of age, long before the appearance of macroorchidism. IGSF1 deficiency is characterized by central hypothyroidism, delayed pubertal testosterone rise, variable prolactin deficiency, variable GH deficiency, metabolic syndrome [104, 122, 123]. Café-au-lait skin pigmentation and precocious puberty can suggest McCune-Albright syndrome [109].

#### Diagnostic evaluation

##### Suggestions and recommendations

**R4.1** We recommend assessing macroorchidism with a complete physical examination, and to use Prader orchidometer to evaluate testicular volume compared with the reference percentiles for age. (1, ⊕  ⊕  ⊕ O).

**R4.2** We recommend testicular ultrasound to rule out testicular lesions and to better assess testicular volume. (1, ⊕  ⊕  ⊕ ⊕).

**R4.3** We suggest evaluating laterality, age at onset and onset time and eventual associate conditions such as mental retardation, café-au-lait skin pigmentation, precocious puberty, phenotypic abnormalities, signs of hypothyroidism or CAH to guide differential diagnosis. (2, ⊕  ⊕  ⊕ O).

**R4.4** We suggest measuring LH, FSH, early morning Testosterone, TSH and FT4 in children and adolescents with macroorchidism. (2, ⊕  ⊕ OO).

##### Evidence

Literature on macroorchidism is limited to case reports and case series in the different clinical context. The assessment of the condition is always performed with physical examination. Testicular ultrasound is the gold standard technique to exclude testis nodules [56]. Outside the context of testicular masses there is not enough evidence on the role of testicular ultrasound features in describing marcoorchid testicles [109, 110]. Testicular function is usually normal [124]. In IGSF1 deficiency, FSH is increased with normal Inhibin B, LH and testosterone can be reduced at the beginning of pubertal development, while thyroid function assessment always shows central hypothyroidism (low/normal TSH and low FT4) [119, 125].

##### Remarks

Macroorchidism should be assessed by physical examination using Prader orchidometer and compared with a table of percentiles for age. Ultrasound is recommended to exclude testicular nodules and for a better assessment of testicular volume. A complete medical history and physical examination is mandatory to address the suspicious of genetic syndromes. FSH, TSH and FT4 assessment is encouraged if no genetic mutation is already known.

#### Therapeutic management

Due to heterogeneity of the macroorchidism pathophysiology, patients’ management should refer to specific underlying diseases, bearing in mind that no effect on testicular volume is expected, since macroorchidism is more a clinical presentation than a pathological condition. In case of finding of testicular lesions, as for adults, specific treatment is recommended [126].

### Precocious puberty

#### Definition and epidemiology

Precocious puberty is defined as the development of secondary sexual characteristics before 8 years of age in girls and 9 years in boys [127]. The estimated incidence in American girls is 1 in 5000–10000 [128]. An observational study from Spain reported an incidence of central precocious puberty between 0.02 and 1.07 cases per 100 000 persons/year [129]. The prevalence is sexually dimorphic, being higher in girls than in boys (15–20 girls for every boy) who more often show an organic cause [130].

#### Pathophysiology

Precocious puberty is classified into two major categories based on the aetiology: Central Precocious puberty (CPP), or GnRH dependent precocious puberty, is secondary to the early activation of the hypothalamus-pituitary–gonadal (HPG) axis [131, 132]. The most relevant causes are reported in Table 1. Peripheral Precocious Puberty (PPP), or GnRH independent precocious puberty**,** is due to the increase of sex steroids from endogenous or exogenous sources, independently of HPG axis activity [133]. It is less common than CPP and the most common causes in boys are reported in Table 1.

**Table 1:** Causes of Precocious puberty

*Central Precocious puberty (CPP)* A) CNS congenital alterations: • Hypothalamic hamartoma • Suprasellar arachnoid cysts • Glioma or neurofibromatosis type 1 • Tuberous sclerosis • Hydrocephalus • Septo-optic dysplasia • Chiari II malformations and myelomeningocele B) CNS acquired alterations: • Tumours: astrocytoma, ependymoma, pinealoma, hypothalamic and optic glioma, craniopharyngioma, dysgerminoma (non-HCG secreting), meningioma • Injury: trauma, cranial irradiation, cerebral palsy, infections • Genetics: loss of function mutation of the MKRN3 gene, gain of function mutation of kisspeptin (KISS1) and its receptor (KISSR) genes, chromosomal abnormalities (Latronico 2016) • Environmental: adopted children, early exposure to sex steroids
*Causes of peripheral precocious puberty (PPP)* • Congenital adrenal hyperplasia (CAH) • McCune-Albright syndrome • Testosterone producing tumours ▪ Adrenal carcinoma or adenoma ▪ Leydig cell tumour • Gonadotropin /HCG producing tumours o Dysgerminoma o Choriocarcinoma o Hepatoblastoma o Chorionepitelioma o Teratoma o Gonadoblastoma • Familial male-limited precocious puberty (testotoxicosis due to germline activating mutation of the LH receptor gene) • Exposure to exogenous sex steroids • Hypothyroidism (Van Wyk-Grumbach syndrome)

#### Clinical picture

In boys, testicular volume equal or greater than 4 mL is the first sign of puberty in CPP whereas a testicular volume less than 4 ml in presence of signs of puberty suggests PPP. Pubic and axillary hair, body odor, hyperpigmentation of penis and scrotum, accelerated growth and advanced bone age are the clinical signs of precocious puberty. Skin examination is necessary for detection of facial acne or oily skin and cutaneous pigmentation (café-au-lait spots). Unilateral testicular enlargement, or the detection of testicular nodules on palpation, suggests a testicular tumor.

#### Diagnostic evaluation

##### Suggestions and recommendations

**R5.1** We recommend in a boy younger than 9 years measuring testicular volume with Prader’s orchidometer and to compare the signs of puberty with the Tanner’s staging method. (1, ⊕  ⊕  ⊕ ⊕).

**R5.2** We recommend performing left hand and wrist X-rays for bone age assessment in all boys with precocious puberty. (1, ⊕  ⊕  ⊕ ○).

**R5.3** We recommend testing all patients with high suspicion for precocious puberty with GnRH stimulation test, basal LH value being less reliable to exclude precocious puberty. (1, ⊕  ⊕  ⊕ ○).

**R5.4** We recommend performing brain MRI in all boys with CPP. (1, ⊕  ⊕  ⊕ ⊕).

**R5.5** We recommend measuring early-morning testosterone, dehydroepiandrosterone sulphate (DHEA-S), 17-OH-progesterone and hCG in boys with suspicion for PPP. (1, ⊕  ⊕  ⊕ ○).

**R5.6** We recommend ultrasound of testes in boys with PPP and testosterone excess and brain MRI in all boys with PPP associated with detectable hCG. (1, ⊕  ⊕  ⊕ ○).

**R5.7** We suggest genetic investigations in boys with CPP and a clear family history of CPP. (2, ⊕  ⊕ ○○).

##### Evidence

Advanced bone age is a hallmark of precocious puberty [134]. The bone age of patients with precocious puberty is generally advanced by 2 years or more compared to chronological age. However, the absence of advanced bone age is not a reason to discontinue follow-up assessment when increased growth velocity and other clinical signs of progressive puberty are present [135].

Careful history, physical exam and growth velocity assessment should guide the evaluation [127]. The child with bilateral enlarged testicles, for example, is most likely to have central precocious puberty, FSH causing the expansion of seminiferous tubule volume. Conversely, the child with clear signs of puberty associated with prepubertal sized testicles is more likely to have PPP, and the child with unilateral testicular enlargement may have a testicular tumour. Testing should be directed accordingly. The gold-standard for biochemical diagnosis of CPP is the assessment of gonadotropins, mainly LH, after stimulation with exogenous GnRH or GnRH agonists. A LH peak response to GnRH equal or greater than 5 mU/mL is consistent with CPP, whereas FSH levels are of limited utility [136]. With the development of laboratory methods based on monoclonal antibodies which have higher sensitivity and specificity than radioimmunoassay methods, baseline random LH assessment has been proposed to evaluate the activation of the gonadotropic axis thus avoiding the need to perform a GnRH stimulation test. However, several studies have shown that the diagnostic value of basal LH is variable, with sensitivity ranging from 60 to 100% [137].

Once the diagnosis of CPP is made, brain MRI scan is mandatory to exclude the presence of congenital or acquired underlying pathology. Most organic causes of CPP are congenital or acquired CNS alterations and even some cases of PPP may be secondary to hCG secreting brain tumour (i.e., dysgerminoma).

Gain-of-function mutations in the genes encoding Kisspeptin and Kisspeptin receptor (*KISS1 KISS1R*) and loss-of-function mutations encoding Makorin ring finger 3 (*MKRN3)* and Delta Like Non-Canonical Notch Ligand 1 (*DLK1)* have been shown in patients with CPP [132]. *MKRN3* mutations are now the most common genetic defect associated with CPP, with an overall frequency of ~ 10% (up to 46% in cases of familial CPP) [132].

In PPP both LH and FSH are suppressed, and androgens are elevated. DHEAS is used to screen for adrenal tumours or adrenal pathology whereas 17-hydroxyprogesterone is used to screen for congenital adrenal hyperplasia secondary to 21-hydroxylase deficiency [138]. High levels of testosterone suggest a testicular tumour. Testicular tumours may be too small to palpate, and ultrasound scan is useful in unexplained PPP cases and may reveal impalpable Leydig cell tumours.

Once the diagnosis of PPP is made, the diagnostic workup will require appropriate imaging and, often, biopsy of the suspected lesion.

##### Remarks

The development of laboratory methods that make use of monoclonal antibodies such as immuno- fluorometric, immunochemiluminometric, and electro-chemiluminometric assays has led some authors to suggest that baseline random LH could be used to assess the activation of the gonadotropic axis, avoiding the need for testing of the GnRH-stimulated LH concentration. However, determining the diagnostic cut-off for the basal LH level is difficult because of a lack of normative data and variability among assays, ranging from 0.1 to 1.5 IU/L.

#### Therapeutic management

#### Central precocious puberty

##### Suggestions and recommendations

**R5.8** We recommend GnRH agonist therapy as the standard of care in children with CPP. GnRHa therapy should be started soon if the child is at or beyond Tanner stage III, particularly when skeletal maturation is advanced of 2 or more years. (1, ⊕  ⊕  ⊕ ○).

**R5.9** We recommend, during treatment, monitoring of pubertal progression, growth velocity every 3 to 6 months, and skeletal maturation every 6 to 12 months. (1, ⊕  ⊕ ○○).

**R5.10** We suggest discontinuing GnRH agonist treatment between 13·0 and 13·5 years of bone age. (2, ⊕ ○○○).

##### Evidence

Long-acting GnRH agonists have been the gold-standard treatment since the mid-1980s [136]. The two main aims of such therapy are to preserve adult height and to prevent psychosocial difficulties. The most important clinical criterion for starting a child on GnRHa treatment is the documented progression of pubertal development, which is based on the recognition that many patients with CPP have a slowly progressive or non-progressive form and achieve adult height within their target range without therapy [139, 140].

GnRH agonists continuously stimulate the pituitary gonadotropin secreting cells thus desensitizing them and reducing LH release and, to a lesser extent, FSH. Several GnRH agonists are available in various depot forms, and their approval for use and recommended doses in precocious puberty vary in different countries. All available GnRHa are effective despite their different routes of administration, dosing, and duration of action. GnRHa induce the regression of pubertal symptoms, reduce growth velocity, and decrease bone-age advancement.

No randomised controlled trials have assessed long-term outcomes after GnRH agonist treatment. Few controlled prospective studies have been performed in children, and many conclusions rely on expert opinions. For example, height outcomes have been assessed mainly by comparisons between achieved adult height and predicted height before treatment [139–141]. Therefore, the evidence supporting the use of GnRH agonists in all children with CPP is weak. The efficacy of such therapy in increasing adult height is undisputed only in early-onset CPP. The real impact of therapy on psychosocial wellbeing needs to be ascertained with rigorously designed longitudinal studies.

GnRH agonists are generally well tolerated and effective. Monitoring of such therapy should consist of assessment of pubertal stage and growth velocity every 3 to 6 months with periodic (every 1–2 years) evaluation of skeletal maturation. Progression of pubertal signs, high growth velocity and advancement of bone age indicate poor compliance, treatment failure, or incorrect diagnosis, demanding further assessment. Regular monitoring of LH and testosterone levels during GnRH agonist therapy has been proposed and suppression of LH to less than 4·5 IU/L during GnRH stimulation test is considered an acceptable target in patients on GnRH agonist therapy. However, an adequate clinical and anthropometric evaluation is a reliable mirror of the endocrine status and may avoid blood tests which should be reserved to patients with poor response to therapy.

The optimal time to discontinue GnRH agonist treatment is difficult to fix because of variability in chronologic age, bone age and degree and duration of secondary sexual characteristics prior to initiation of therapy. In addition, data from boys with CPP are extremely limited. Pubertal signs reappear within months after cessation of GnRH agonist treatment. It could be reasonable decide when to stop therapy according to patient and family preference with the intent of synchronizing pubertal development with the patient’s peers.

##### Remarks

GnRH agonists are usually well tolerated. Treatment can be associated with headaches, rash, gastrointestinal complaints. These adverse effects are generally transient and resolve spontaneously or with symptomatic treatment. Local complications such as sterile abscesses can result in a loss of efficacy, and anaphylaxis has been described in rare cases.

Long-term fertility has not been fully assessed, but preliminary data showed normal reproduction function. Bone mineral density can decrease during GnRH agonist therapy, but subsequent bone mass accrual is preserved, and peak bone mass is not affected. A potential negative effect on BMI was postulated but the available data suggest that long-term GnRH agonist treatment does not cause or aggravate obesity.

#### Peripheral precocious puberty

##### Suggestions and recommendations

**R5.11** We recommend treating (surgically or medically) the underlying cause of PPP. (1, ⊕  ⊕  ⊕ ⊕).

**R5.12** We recommend combination therapy with antiandrogen and third-generation aromatase inhibitor in severe cases of PPP with androgen excess. (1, ⊕ ○○○).

##### Evidence

GnRH-independent forms of precocious puberty are a spectrum of many different conditions often associated with androgen hypersecretion secondary to tumours. Together with the removal of the neoplasm responsible for androgen excess, combination therapy with a potent antiandrogen, bicalutamide, and a third-generation aromatase inhibitor is effective in reducing virilization, growth rate, skeletal maturation and improving adult height. However, no large-scale longitudinal study has been conducted so far and most available data come from case reports. It should be also remarked that these treatments are mostly off-label in several countries. Treatment of PPP should be personalized according to the aetiology. Children with congenital adrenal hyperplasia, for example, will benefit from glucocorticoid therapy. In poorly controlled patients with long-lasting hyperandrogenism associated with bone age advancement secondary CPP may occur thus requiring treatment with GnRH agonists [142, 143].

##### Remarks

PPP is much less common than CPP and poses ongoing challenges in the pursuit of the optimal clinical management. Collaborative multicentre long-term clinical trials are needed to achieve sufficient evidence to guide to the appropriate therapy and to unveil the ultimate outcomes for these children.

### Delayed puberty

#### Epidemiology

Delayed puberty (DP) in male subjects is defined as the failure to enter puberty by the age of 14 years or to complete the process, with a prolonged tempo of pubertal development (above 5.2 years) [144, 145]. It is a common condition affecting up to 2% of adolescents [146, 147]. DP is particularly prevalent in boys and for this reason is across-the-board of andrology and both pediatric and adult endocrinology. In boys is mainly related (63–64% of cases) to a self-limited condition called Constitutional Delay of Growth and Puberty (CDGP) [148–151] whereas the remaining part consists of pathological conditions determining a functional (FHH, 18–20% of DP) or persistent hypogonadism (≈15%) [146–149, 152–154].

#### Pathophysiology

CDGP is the most frequent form of DP and represent a paraphysiological condition which is self-limited with a common familial component [151, 155]. DP due to hypogonadism conditions can be classified according to the origin of the failure of the hormonal axis in primary or hypergonadotropic hypogonadism (gonadal origin) and central, or hypogonadotropic hypogonadism (pituitary/hypothalamic origin). Primary hypogonadism can be caused by acquired gonadal damage (including radio- and chemo-therapy), congenital causes such as chromosomal abnormalities (Klinefelter syndrome, KS) or other genetic conditions impairing gonadal development, gonadotropin receptors or steroidogenic enzymes [156].

Central hypogonadism can be distinguished in functional (usually reversible) and organic forms. FHH is usually determined by a dysregulation of the hypothalamic-pituitary–gonadal axis caused by metabolic and/or inflammatory condition (which include malnutrition, chronic illnesses, inflammatory diseases such as coeliac disease and inflammatory bowel diseases, and anorexia nervosa). Organic forms include acquired structural (i.e. invasive, infiltrative, inflammatory, ischemic or even iatrogenic lesions to the pituitary and/or the hypothalamus) and congenital alterations such as multiple pituitary hormone defects (MPHD) and congenital isolated hypogonadotropic hypogonadism (CHH), which represent the main differential diagnosis of CDGP [144, 150, 151, 154, 155, 157].

#### Clinical picture

**Fig. 1:**
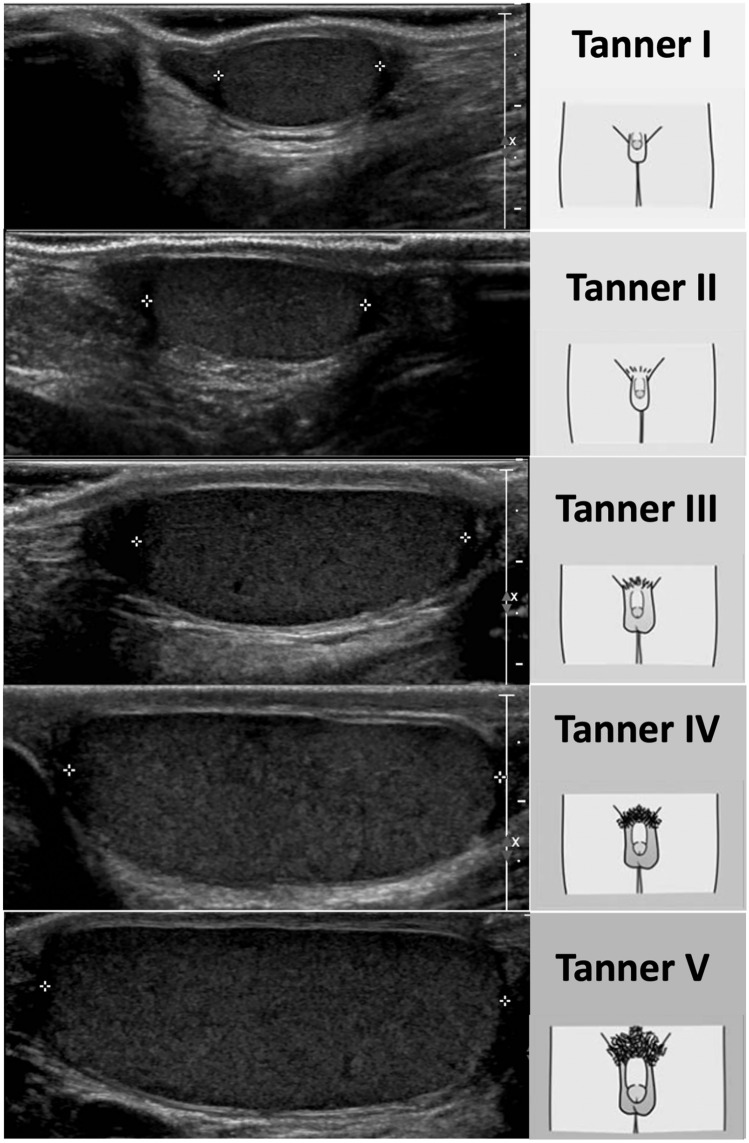
Normal testicular ultrasound according to Tanner stages

The clinical presentation of DP in boys is the failure to start and/or complete physiologic testicular development (Fig. 1), the lack of growth spurt and, usually, the lack of signs of virilization such as pubic hair and secondary sexual characteristics. Bone age is always delayed of at least 1 year in CDGP and is extremely helpful in guiding the diagnostic work-up. Boys with CGDP are usually referred to the specialist for short stature secondary to the delayed pubertal growth spurt but their slow “tempo” of growth does not impair the achievement of a normal adult height corresponding to their genetic growth potential (midparental height). Olfactory defects, hearing impairment, midline defects and kidney/teeth abnormalities as well as other syndromic features can be present if the delay of puberty is part of a more complex disease [151, 158].

#### Diagnostic evaluation

##### Suggestions and recommendations

**R6.1** We recommend an expert evaluation of pubertal development in any boy presenting with DP defined by no testicular enlargement equal or above 4 mL (measured with Prader’s orchidometer) at the age of 14 years, or the failure to progress adequately in his pubertal development.

(1, ⊕  ⊕  ⊕ ⊕).

**R6.2** We recommend measuring LH, FSH, early-morning total testosterone (T) levels and assessing bone age with left hand and wrist radiography in all subjects with DP. (1, ⊕  ⊕  ⊕ ⊕).

**R6.3** We recommend performing karyotype and testicular ultrasound (US) in patients with high gonadotropin levels to rule in/out Klinefelter syndrome and/or testicular damage. (1, ⊕  ⊕  ⊕ ⊕).

**R6.4** We suggest adopting a “wait and see” strategy evaluating the patient after six months in all subjects with DP and Tanner stage 1, LH levels above 0.2 IU/L but below the upper limit range, T above 0.7 nmol/L and bone age below 13 years. (2, ⊕  ⊕ ○○).

**R6.5** We suggest testing with GnRH stimulation test all patients with DP, LH levels below 0.2 IU/L and T below 0.7 nmol/L or no clinical (no Tanner stage 2 achievement) and hormonal (LH and T increase) progressions after six months of “wait and see” strategy. (2, ⊕  ⊕ ○○).

**R6.6** We recommend excluding other diseases causing acquired secondary hypogonadism in all subjects with DP, low T and low gonadotropins and not responsive to GnRH test or bone age above 13 years. (1, ⊕  ⊕  ⊕ ○).

**R6.7** We recommend referring patients with a diagnosis of CHH to a dedicated center to perform a genetic analysis and family counselling. (1, ⊕  ⊕ ○○).

##### Evidence

The primary goal of the clinician is to define a true DP, which could be a failure to start or to complete the process in due time, and its underlying cause. The definition of a standard pubertal timing in male is defined by a testicular volume equal or above 4 mL before the age of 14 years [158, 159] and the normal duration of the entire process should not exceed 5.2 years [144, 145]. Nonetheless, also the absence of growth spurt by the age of 16 is a sign of DP [160].

Accurate clinical history collection is mandatory including a detailed family history for pubertal or endocrinological disorders. Yet, both CHH and CDGP patients show positive familiar history which can also be shared between them with up to 10% of family members of CHH patients showing a delayed puberty [161].

At the physical examination, it is important to evaluate the Tanner stage of the patient [158, 159], his growth and the so called “red flags” reported in Table 2, as hallmarks of likely CHH [3, 150, 151, 162, 163]. Furthermore, the interruption of pubertal development after its onset is suggestive for hypogonadism with a bone age above 13 years [158, 164].

**Table 2:** The “red flags” suggesting a diagnosis of CHH

Red flags
Cryptorchidism
Microrchidism (< 1.1 mL)
Micropenis (according SD for age) doi:10.1001/archpedi.1943.02010160019003
Hypospadias
Hypo-/anosmia
Hearing defects
Midline defects (i.e., cleft lip and/or palate)
digital abnormalities
Bimanual synkinesis or “mirror movements”
Dental dysgenesis/agenesis

High gonadotropin levels, especially along with low T, are clearly diagnostic of primary (hypergonadotropic) hypogonadism [146]. Among the main causes of hypergonadotropic hypogonadism there are: anorchia, testicular damages including traumatic, iatrogenic (i.e. chemo-and/or radiotherapy), infective ones and congenital causes such as KS (although patients with KS usually have a spontaneous onset of puberty that may not reach full virilization at its end). A karyotype (for KS) and testicular ultrasound scan (for gonadal damage), along with personal history, are necessary for the diagnosis [158, 165]. If negative, the patient should be referred to a tertiary centre to evaluate other genetic conditions and complex syndromes.

At the beginning of pubertal development LH and FSH are very low and the use of ultransensitive assays (i.e., detection limit < 0.1 IU/L) are required to discriminate between prepubertal from pubertal children based on their concentration [166]. At this respect, reference ranges for both gonadotropins using ultrasensitive assays are available for all ages including the pubertal transition period [167]. LH levels above 0.2 IU/L are suggestive for the beginning of puberty [168]. Similarly, early-morning T levels, measured with specific and ultrasensitive assays, above 0.7 nmol/L are a biochemical sign of the beginning of puberty which will be followed by pubertal signs within 12–15 months [169]. If T and gonadotropins are below these thresholds, the finding is consistent with CDGP or hypogonadotropic hypogonadism. GnRH test measures the previous stimulation of the pituitary gonadotropes from GnRH. A boy with DP responding to GnRH test (i.e. LH peak > 5 IU/L) [170], will likely start puberty within 1 year [168, 171].

##### Remarks

Although the expert evaluation of pubertal development should be started when no signs of pubertal development are present by the age of 14 years in boys, we should anticipate this assessment at the age of 9 years in all children with documented risk of persistent hypogonadism.

It should be emphasized that bone age should be assessed by an experienced physician (pediatric endocrinologist, endocrinologist, radiologist) or by a validated digital system.

It is worth mentioning that GnRH testing is useful to unmask the beginning of puberty, but not to perform a differential diagnosis between CDGP and CHH [150, 171]. Indeed, in patients with initial elevation of LH and T or testing positive to GnRH stimulation test, it might be considered adopting a “wait and see” strategy for other six months, starting further specific investigation if there is no pubertal progression during this period.

In patients presenting a bone age discordant with the hormone levels or with no signs of pubertal onset, it is important to exclude all the possible causes of acquired hypogonadotropic hypogonadism including both functional (Table 3) and organic/structural forms. A short child with DP should be screened for excluding celiac disease (anti-transglutaminase antibodies), hypothyroidism (FT4 and TSH), GH deficiency (IGF1) and inflammatory bowel disease (fecal calprotectin). In case of subnormal or low normal range IGF1 levels, boys with DP and growth impairment should be tested for GHD using specific dynamic evaluations according to the indication of the consensus of the GH society [172, 173] after priming with sex steroids [174–178].

**Table 3:** causes of functional hypogonadotropic hypogonadism (FHH)

Causes of FHH
Malnutrition and Anemia
Anorexia nervosa
Low energy availability/relative energy deficit
Coeliac disease
IBD
Systemic inflammatory disease
Obesity
Other chronic illnesses
Other endocrine diseases (i.e. hyperPRL, cushing, etc.)
Drugs

It is also important to evaluate pituitary function (including cortisol, ACTH, FT4, FT3, IGF1 levels, prolactin) and perform brain MRI scan with pituitary and olfactory tract assessment in all subjects with a suspected diagnosis of hypogonadotropic hypogonadism.

Genetic analysis (e.g., Targeted-Next Generation Sequencing or Whole Exome Sequencing techniques) could be helpful to diagnose CHH if a known causative gene variant is found [3, 179, 180]. Genetic analyses and counselling should be performed in tertiary referral center due to the expertise needed for genetic counselling and rare variants interpretation [179, 180].

#### Treatment

##### Suggestions and recommendations

**R6.8** We recommend starting the proper treatment once the diagnosis of hypogonadism is well-established. (1, ⊕  ⊕  ⊕ ⊕).

**R6.9** We recommend starting treatment with intramuscular or transdermal testosterone once the diagnosis of hypergonadotropic hypogonadism is confirmed. (1, ⊕  ⊕  ⊕ ⊕).

**R6.10** We suggest discussing the possible initial treatment with gonadotropins in patients with hypogonadotropic hypogonadism. (2, ⊕  ⊕  ⊕ ○).

**R6.11** We suggest using low-dose sex steroids for three to six months, which can be repeated for other three to six months, to distinguish DP due to CDGP from CHH and to manage patients’ psychological discomfort. (2, ⊕  ⊕  ⊕ ○).

**R6.12** We recommend treating the underlying causes of FHH and to consider a medical treatment for puberty induction whenever this is not feasible. (1, ⊕  ⊕  ⊕ ○).

##### Evidence

It is important to treat persistent forms of hypogonadism once diagnosed. It is worth noting that the acceleration of bone maturation and the risk of reducing near adult height are increased when treatment is started too early. Treatment from the age of 12 years has been shown to be safe in patients with a high risk of hypogonadism [181–185].

Testosterone is the only effective treatment for patients with hypergonadotropic hypogonadism. The evidence regarding the induction of puberty using testosterone esters with lower half-life (monthly or weekly testosterone esters) are stronger than the ones regarding either testosterone gel or long half-life intramuscular testosterone undecanoate. However, transdermal formulations seem to be a more viable and practical solution compared to long acting undecanoate. Current formulations of oral testosterone are not recommended due to the low half-life and the high hepatic concentrations [186].

Gonadotropins have shown potential efficacy for both reproductive and non-reproductive outcomes in patients with hypogonadotropic hypogonadism [187]. Treatment with both gonadotropins and testosterone should be discussed with these patients and their family, providing a balanced counselling about benefits and drawbacks [184, 186].

The approach to the boy with high suspicion of CGDP can be “wait and see” or based on therapy with low-doses of testosterone [150, 151, 158]. A trial with low-doses of androgens has proved to increase the growth rate of these patients, as well as improve their quality of life and psychological well-being [188–190]. Moreover, recent evidence suggests a potential impact on near adult height in CDGP boys and on body proportion of CHH patients treated with low-dose sex steroids [148, 174, 187, 190]. This low-dose testosterone therapy (50 mg per month for 3–4 months) could be proposed to patients with decreased self-esteem, psychosocial difficulties in the interaction with peers [148, 158, 188–191]. This approach has been shown to be safe not affecting bone age maturation and adult height [188–190], and it could be useful for preventing a lower bone mass in adulthood [192].

##### Remarks

The treatment with low-dose sex steroids can be performed with both intramuscular esters and transdermal formulations, as reported in Table 4.

**Table 4:** therapeutic schemes for low doses of sex steroids

Formulation	Dosing and administration
Testosterone enanthate	50 mg i.m. monthly
Testosterone gel 2%	10 mg every second day

It should also be noted that patients who do not achieve spontaneous puberty after one year of treatment with low-dose sex steroids are to be considered hypogonadotropic hypogonadism and should undergo careful evaluation of pituitary function [165].

Finally, in patients with a diagnosis of DP undergoing a trial with low-dose sex steroids, if growth spurt does not occur during the treatment, FHH should be reconsidered.

## Supplementary Information

Below is the link to the electronic supplementary material.

Supplementary file1 (DOCX 26 KB)
