# Supplementary material for: “Management of andrological disorders from childhood and adolescence to transition age: guidelines from the Italian Society of Andrology and Sexual Medicine (SIAMS) in collaboration with the Italian Society for Pediatric Endocrinology and Diabetology (SIEDP)—Part-1”
Source: J Endocrinol Invest. 2024 Aug 10;48(1):1–22. doi: 10.1007/s40618-024-02435-x (PMC11729124; doi:10.1007/s40618-024-02435-x)
Supplement: Supplementary file 1 — Supplementary file1 (DOCX 26 KB) [file 40618_2024_2435_MOESM1_ESM.docx]

**List of suggestions and recommendations**

**1. Varicocele**

**R1.1** We recommend assessing varicocele by physical examination in recumbent and standing position. (1, ⊕⊕⊕○)

**R1.2** We suggest using color Doppler ultrasound to further characterize and grade the varicocele. (2, ⊕⊕○○)

**R1.3** We recommend using ultrasound to accurately assess testicular hypotrophy or asymmetry. (1, ⊕⊕○○)

**R1.4** We suggest offering treatment in adolescents with testicular hypotrophy or asymmetry (difference >20%). (2, ⊕⊕○○)

**R1.5** We suggest considering pain and discomfort for decision making about varicocele treatment, despite evidence is limited. (Expert Opinion)

**R1.6** We suggest lymphatic sparing surgery or percutaneous embolization, although, at present, no technique has demonstrated to be better. **(**2, ⊕⊕⊕○**)**

**R1.7** We suggest only monitoring for patients not candidate for surgical treatment. **(**Expert Opinion**)**

**2. Gynecomastia**

**R2.1** We suggest that the initial screening to rule out lipomastia, obvious breast cancer, or testicular cancer might be performed by a pediatrician, a general practitioner, or another non-specialist. (2, ⊕⊕○○)

**R2.2** We recommend that in those cases where a thorough diagnostic workup is warranted, it should be performed by a specialist in endocrinology/pediatric endocrinology/andrology. (1, ⊕⊕⊕○)

**R2.3** We recommend testis and breast US to rule out possible tumors. (1, ⊕⊕⊕○)

**R2.3** We do not recommend medical therapy for pubertal gynecomastia, which resolves spontaneously within 24 months in more than 90% of cases. (1, ⊕⊕○○)

**R2.4** We recommend withdrawal of interfering drugs, whenever feasible, treatment of concomitant disorders, and discontinuation of substances of abuse or inducing drugs, before considering a specific treatment for gynecomastia. (1, ⊕⊕⊕○)

**R2.5** We suggest surgical treatment only for patients with long-lasting gynecomastia, which does not regress spontaneously, or which causes considerable psychosocial and psychological distress, after puberty completion. (2, ⊕⊕○○)

**3. Fertility preservation**

**R3.1** We recommend testicular physical examination and pubertal stage evaluation in all pediatric patients to guide towards the appropriate FP option. (1, ⊕⊕⊕⊕)

**R3.2** We suggest providing adequate counseling on FP to these patients and their parents. (Expert Opinion)

**R3.3** We recommend sperm cryopreservation in all young boys able to collect semen and in which it is possible to retrieve mature sperm. (1, ⊕⊕⊕⊕)

**R3.4** We suggest discussing testicular biopsy with tissue banking in case of prepubertal or azoospermic subjects. (2, ⊕⊕○○)

**4. Macroorchidism**

**R4.1** We recommend assessing macroorchidism with a complete physical examination, and to use Prader orchidometer to evaluate testicular volume compared with the reference percentiles for age. (1, ⊕⊕⊕O)

**R4.2** We recommend testicular ultrasound to rule out testicular lesions and to better assess testicular volume. (1, ⊕⊕⊕⊕)

**R4.3** We suggest evaluating laterality, age at onset and onset time and eventual associate conditions such as mental retardation, café-au-lait skin pigmentation, precocious puberty, phenotypic abnormalities, signs of hypothyroidism or CAH to guide differential diagnosis. (2, ⊕⊕⊕O)

**R4.4** We suggest to dose LH, FSH, Testosterone, TSH and FT4 in children and adolescents with macroorchidism. (2, ⊕⊕OO)

**5. Precocious puberty**

**R5.1** We recommend in a boy younger than 9 years measuring testicular volume with Prader’s orchidometer and to compare the signs of puberty with the Tanner’s staging method. (1, ⊕⊕⊕⊕)

**R5.2** We recommend performing left hand and wrist X-rays for bone age assessment in all boys with precocious puberty. (1, ⊕⊕⊕○)

**R5.3** We recommend testing all patients with high suspicion for precocious puberty with LHRH stimulation test, basal LH value being less reliable to exclude precocious puberty. (1, ⊕⊕⊕○).

**R5.4** We recommend performing brain MRI in all boys with CPP. (1, ⊕⊕⊕⊕)

**R5.5** We recommend measuring early-morning testosterone, dehydroepiandrosterone sulphate (DHEA-S), 17-OH-progesterone and hCG in boys with suspicion for PPP. (1, ⊕⊕⊕○).

**R5.6** We recommend ultrasound of testes in boys with PPP and testosterone excess and brain MRI in all boys with PPP associated with detectable hCG. (1, ⊕⊕⊕○)

**R5.7** We suggest genetic investigations in boys with CPP and a clear family history of CPP. (2, ⊕⊕○○)

**R5.8** We recommend GnRH agonist therapy as the standard of care in children with CPP. GnRHa therapy should be started soon if the child is at or beyond Tanner stage III, particularly when skeletal maturation is advanced of 2 or more years. (1, ⊕⊕⊕○)

**R5.9** We recommend, during treatment, monitoring of pubertal progression, growth velocity every 3 to 6 months, and skeletal maturation every 6 to 12 months. (1, ⊕⊕○○)

**R5.10** We suggest discontinuing GnRH agonist treatment between 13·0 and 13·5 years of bone age. (2, ⊕○○○)

**R5.11** We recommend treating (surgically or medically) the underlying cause of PPP. (1, ⊕⊕⊕⊕)

**R5.12** We recommend combination therapy with antiandrogen and third-generation aromatase inhibitor in severe cases of PPP with androgen excess. (1, ⊕○○○)

**6. Delayed puberty**

**R6.1** We recommend an expert evaluation of pubertal development in any boy presenting with DP defined by no testicular enlargement equal or above 4 mL (measured with Prader’s orchidometer) at the age of 14 years, or the failure to progress adequately in his pubertal development.
(1, ⊕⊕⊕⊕)

**R6.2** We recommend measuring LH, FSH, early-morning total testosterone (T) levels and assessing bone age with left hand and wrist radiography in all subjects with DP. (1, ⊕⊕⊕⊕)

**R6.3** We recommend performing karyotype and testicular ultrasound (US) in patients with high gonadotropin levels to rule in/out Klinefelter syndrome and/or testicular damage. (1, ⊕⊕⊕⊕)

**R6.4** We suggest adopting a “wait and see” strategy evaluating the patient after six months in all subjects with DP and Tanner stage 1, LH levels above 0.2 IU/L but below the upper limit range, T above 0.7 nmol/L and bone age below 13 years. (2, ⊕⊕○○)

**R6.5** We suggest testing with LHRH stimulation test all patients with DP, LH levels below 0.2 IU/L and T below 0.7 nmol/L or no clinical (no Tanner stage 2 achievement) and hormonal (LH and T increase) progressions after six months of “wait and see” strategy. (2, ⊕⊕○○)

**R6.6** We recommend excluding other diseases causing acquired secondary hypogonadism in all subjects with DP, low T and low gonadotropins and not responsive to LHRH test or bone age above 13 years. (1, ⊕⊕⊕○)

**R6.7** We recommend referring patients with a diagnosis of CHH to a dedicated center to perform a genetic analysis and family counselling. (1, ⊕⊕○○)

**R6.8** We recommend starting the proper treatment once the diagnosis of hypogonadism is well-established. (1, ⊕⊕⊕⊕)

**R6.9** We recommend starting treatment with intramuscular or transdermal testosterone once the diagnosis of hypergonadotropic hypogonadism is confirmed. (1, ⊕⊕⊕⊕)

**R6.10** We suggest discussing the possible initial treatment with gonadotropins in patients with hypogonadotropic hypogonadism. (2, ⊕⊕⊕○)

**R6.11** We suggest using low-dose sex steroids for three to six months, which can be repeated for other three to six months, to distinguish DP due to CDGP from CHH and to manage patients’ psychological discomfort. (2, ⊕⊕⊕○)

**R6.12** We recommend treating the underlying causes of FHH and to consider a medical treatment for puberty induction whenever this is not feasible. (1, ⊕⊕⊕○)
